# Novelty of digital press stereolithography (DPS) with MIDAS system in crown fabrication: marginal and internal fit evaluation

**DOI:** 10.1186/s12903-025-06818-1

**Published:** 2025-09-26

**Authors:** Cristian Abad-Coronel, S. Martín Proaño, S. Michelle González, L. Jerely Chico, Nancy Mena Córdova, Fabián Rosero, Paulina Aliaga

**Affiliations:** 1 Digital Dentistry and CAD/CAM Materials Research Group, Faculty of Dentistry, Universidad de Cuenca, Cuenca, Ecuador; 2 Department of Prosthodontics, Faculty of Dentistry, Universidad San Francisco de Quito, Quito, Ecuador; 3 Private Practice, Quito, Ecuador

**Keywords:** CAD/CAM, Additive manufacturing, Marginal gap, Internal fit, Digital press stereolithography, Zirconia, 3D printing

## Abstract

**Background:**

The marginal and internal fit of full-coverage crowns is essential for their long-term clinical success. Computer-aided design and manufacturing (CAD/CAM) technologies have enhanced the precision of restorations. However, the performance of emerging three-dimensional (3D) printing systems, such as the Midas system based on digital press stereolithography (DPS), requires further investigation.

**Methods:**

This in vitro study evaluated and compared the marginal, cervical, axial, and occlusal gaps of crowns fabricated using five different materials. A total of forty crowns were fabricated using subtractive milling (Empress CAD, Vita Enamic, Cerasmart, and zirconia; *n* = 10 each), and ten crowns were fabricated using additive 3D printing with the Midas DPS system. A standardized molar preparation was scanned and used to produce fifty resin dies. Crowns were designed using dedicated software, cemented on the dies, and subjected to thermocycling (5000 cycles between 5 °C and 55 °C). Each specimen was sectioned and examined under 40× magnification using a stereomicroscope. A total of 160 gap measurements were recorded for each crown across four anatomical regions. Statistical analysis was performed using the Shapiro–Wilk, Kruskal–Wallis, and Mann–Whitney U tests with a significance level set at 0.05.

**Results:**

All groups exhibited gap values within clinically acceptable ranges. Zirconia crowns demonstrated the lowest mean gaps and variability, especially in the cervical (66.0 micrometers, coefficient of variation: 6.1%) and axial (122.7 micrometers, coefficient of variation: 2.9%) regions. The Midas 3D-printed group presented greater variability, particularly in the occlusal region (211.9 micrometers, coefficient of variation: 52.1%). Statistically significant differences were found in cervical gap values among the materials tested.

**Conclusions:**

Crowns fabricated using the Midas DPS 3D printing system exhibited acceptable adaptation, although with greater variability compared to those produced via subtractive methods. Zirconia demonstrated superior dimensional consistency, supporting its continued use as a reference material. These findings indicate that the Midas system holds promise as a clinically viable alternative, warranting further validation through clinical studies.

## Introduction

Computer-aided design and computer-aided manufacturing (CAD/CAM) technologies have revolutionized the fabrication of dental restorations, leading to a significant rise in their clinical use [1]. These workflows begin with intraoral scanning to capture dental geometry, followed by computer-based design of restorations, prosthetic components, surgical guides, splints, and other devices [2, 3]. The resulting digital files can be stored on cloud-based platforms, enabling efficient communication with dental laboratories and real-time design adjustments [4].

Indirect restorations fabricated using CAD/CAM systems overcome common limitations of direct restorations, such as polymerization shrinkage and difficulties in achieving optimal interproximal contacts. These restorations can be produced via additive (3D printing) or subtractive (milling) methods [5], and must meet four clinical requirements: marginal adaptation, biocompatibility, esthetics, and mechanical resistance [6]. Their strength depends on material composition and can be affected by intraoral conditions including acidic foods, enzymes, and cariogenic biofilms [7, 8].

Subtractive techniques use prefabricated industrial blocks with high structural homogeneity, produced under controlled conditions and high temperatures. Dental ceramics such as zirconia oxide, alumina, and feldspathic porcelain are popular due to their ability to replicate natural tooth function and appearance [9, 10], while also offering biocompatibility, chemical stability, and long-term success [11, 12]. These materials are known for smooth margins, high load-bearing capacity, fatigue resistance, and polishability [13]. Alternatively, polymer-based and hybrid materials allow for faster milling, easier clinical repairs, and reduced risk of marginal fractures [5, 14].

Additive manufacturing, on the other hand, employs photopolymerizable resins that cure layer by layer upon light exposure [15, 16]. These materials, composed of ceramic fillers in organic matrices, are engineered for dimensional stability, mechanical reliability, and biocompatibility. Despite their advantages, they require post-processing steps such as washing, curing, and polishing [17, 18]. The clinical performance of these composites is highly dependent on resin matrix composition, filler content, and silane coupling agents, which influence their fatigue resistance and chemical stability [19].

Among the latest 3D printing technologies is Digital Press Stereolithography (DPS), implemented in the Midas system (MI, SprintRay, San Diego, USA) [20]. This system uses highly viscous, capsule-based resins with elevated ceramic filler content and 385 nm light curing, optimizing filler–matrix interaction and minimizing resin waste. Full crowns can be printed in approximately 10 min.

A critical determinant of success in full-coverage restorations is internal and marginal fit, expressed as the gap between restoration and preparation surfaces. Poor adaptation can lead to microleakage, cement dissolution, secondary caries, sensitivity, and reduced mechanical performance [21]. Additionally, internal voids or porosities in composite materials can compromise properties such as fracture toughness and fatigue resistance [22]. To date, there is limited scientific evidence evaluating the marginal and internal adaptation of restorations fabricated using the MI system with Digital Press Stereolithography (DPS). This study is among the first to investigate the gap behavior of full-coverage crowns produced by this novel 3D printing technology, providing valuable initial insights into its clinical potential. Recent investigations on 3D-printed zirconia and resin-based crowns further support the relevance of this topic. Hassan et al. reported that 3D-printed zirconia crowns exhibited slightly higher marginal and internal gap values compared to milled zirconia, although both remained within clinically acceptable thresholds [23]. Similarly, Elsayed et al. found that milled restorations showed superior adaptation compared to 3D-printed and prefabricated zirconia crowns, though all groups demonstrated clinically acceptable fit values [24]. Additionally, hybrid resin-ceramic materials fabricated via additive manufacturing have shown comparable fit to milled counterparts, reinforcing the potential of high-performance printed materials for definitive restorations [25].

In vitro testing under standardized conditions is essential for evaluating the performance of new CAD/CAM restorative materials. Given the growing number of available materials, more independent studies are necessary. To date, little evidence exists on the marginal and internal fit of crowns produced by the MI-DPS system. This study is one of the first to assess the gap behavior of full crowns fabricated using this novel 3D printing method, providing preliminary data on its clinical potential.

This in vitro study aimed to assess and compare the marginal, cervical, axial, and occlusal gap dimensions of full-coverage crowns fabricated using the new MI-DPS 3D printing system versus subtractive CAD/CAM materials with varying compositions. Given the growing interest in additive manufacturing for definitive restorations, it is essential to evaluate the adaptation of these materials relative to conventional options. The null hypothesis was that there would be no statistically significant differences in gap dimensions among the evaluated CAD/CAM materials.

## Methods

### Sample preparation

 A typodont model with a standardized full-coverage crown preparation of the maxillary first molar was used to fabricate the specimens. The preparation followed specific geometric parameters: 1.5 mm occlusal reduction, 1.5 mm axial reduction, a continuous peripheral chamfer finish line, 10° total occlusal convergence, and rounded internal angles. The indirect restorative materials were divided into five groups (*n* = 10), resulting in a total of 50 crowns. The composition and technical characteristics of the materials used in this study are detailed in Table 1.

**Table 1:** CAD/CAM restorative materials evaluated in the study

CAD/CAM Method	Material	Batch	Manufacturer	Composition
Subtractive	VITA Enamic (VE)	99,430	Vita Zahn Fabrik; Bad Säckingen, Germany	86% ceramic matrix. Pre-sintered and porous compact feldspar. 14% polymeric infiltrate. UDMA and TEGDMA.
Subtractive	EMPRESS CAD (EM)	YBC2DR	Ivoclar Vivadent; Schaan, Liechtenstein	Lithium disilicate feldspathic ceramics.
Subtractive	Cerasmart (CE)	2,112,131	GC Corporation; Tokyo, Japan	Silica nanoparticles (20 nm) and barium glass (300 nm), 71 wt%; Bis-MEPP polymers, UDMA, DMA 29 wt%.
Subtractive	Zirconia ZIRCOSTAR (ZI)	CA1C9818AP	Kerox; Hungary	ZrO2 90.2–94.3%; Y2O3 5.7–9.8%; Al2O3 0.25%; SiO2, Fe2O3, Na2O 0.02%.
Additive	Ceramic Crown (MI)	M24J015	SprintRay; California, USA	Methacrylate and acrylic monomers, photoinitiators, inorganic fillers > 50%.

### Digital workflow

 A prefabricated model was digitized using an intraoral scanner (PrimeScan; Dentsply Sirona, USA). The resulting CAD file in STL format was imported into a 3D printer, and 50 dies were fabricated using a resin-based material (Crown; SprintRay, USA) through a DLP technology printer (Pro S; SprintRay, USA). The printing orientation was automatically determined by the system software (Rayware, SprintRay, USA), and all crowns were printed at a layer thickness of 50 μm. Post-processing, including washing and drying, was carried out using an automated unit (Pro Wash/Dry; SprintRay, USA), followed by light curing in a dedicated post-curing system specific to the resin material (ProCure 2; SprintRay, USA). For the fabrication of the final restorations, the design file was exported in.dxd format via a cloud-based data platform (Connect Case Center; Dentsply Sirona, USA). The crown design was performed using InLab CAD software version 22.1 (Dentsply Sirona, USA), with restoration parameters specified in Table 2.

**Table 2:** Digital parameters of the restorations

Parameter	Value	Unit	Description
Radial spacer	80	µm	Space between the inner surface of the crown and the die wall (axial)
Occlusal spacer	70	µm	Space at the occlusal surface for cement
Strength of occlusal contacts	25	µm	Adjustment intensity for static occlusal contacts
Strength of dynamic contacts	25	µm	Adjustment intensity for dynamic (excursive) occlusal contacts
Minimum radial thickness	800	µm	Minimum material thickness on axial walls
Minimum occlusal thickness	800	µm	Minimum material thickness on the occlusal surface
Edge thickness	100	µm	Minimum thickness at the restoration margin
Width of ramp	50	µm	Width of insertion ramp used in margin design
Angle of ramp	60	°	Insertion ramp angle at the margin

### Fabrication of additive restorations using MIDAS (DPS technology)

The MI additive group (SprintRay, California, USA) was fabricated using a 3D printer (Midas, SprintRay) and a capsule-based ceramic-filled resin designed for definitive restorations (Ceramic Crown, SprintRay, USA), employing the DPS technology. Capsules were pressed in batches of three for 10 min, with one crown obtained from each capsule, resulting in a total of 10 crowns within 40 min. The printed crowns were then manually cleaned using 99% isopropyl alcohol to remove excess resin. Post-curing was performed in an automated chamber (NanoCure; SprintRay, USA) for 3 min, following the manufacturer’s instructions. Final polishing was completed using an extraoral polishing system (AP Polishing and Finishing Kit; Tampa, USA).

### Fabrication of subtractive restorations

**Fig. 1:**
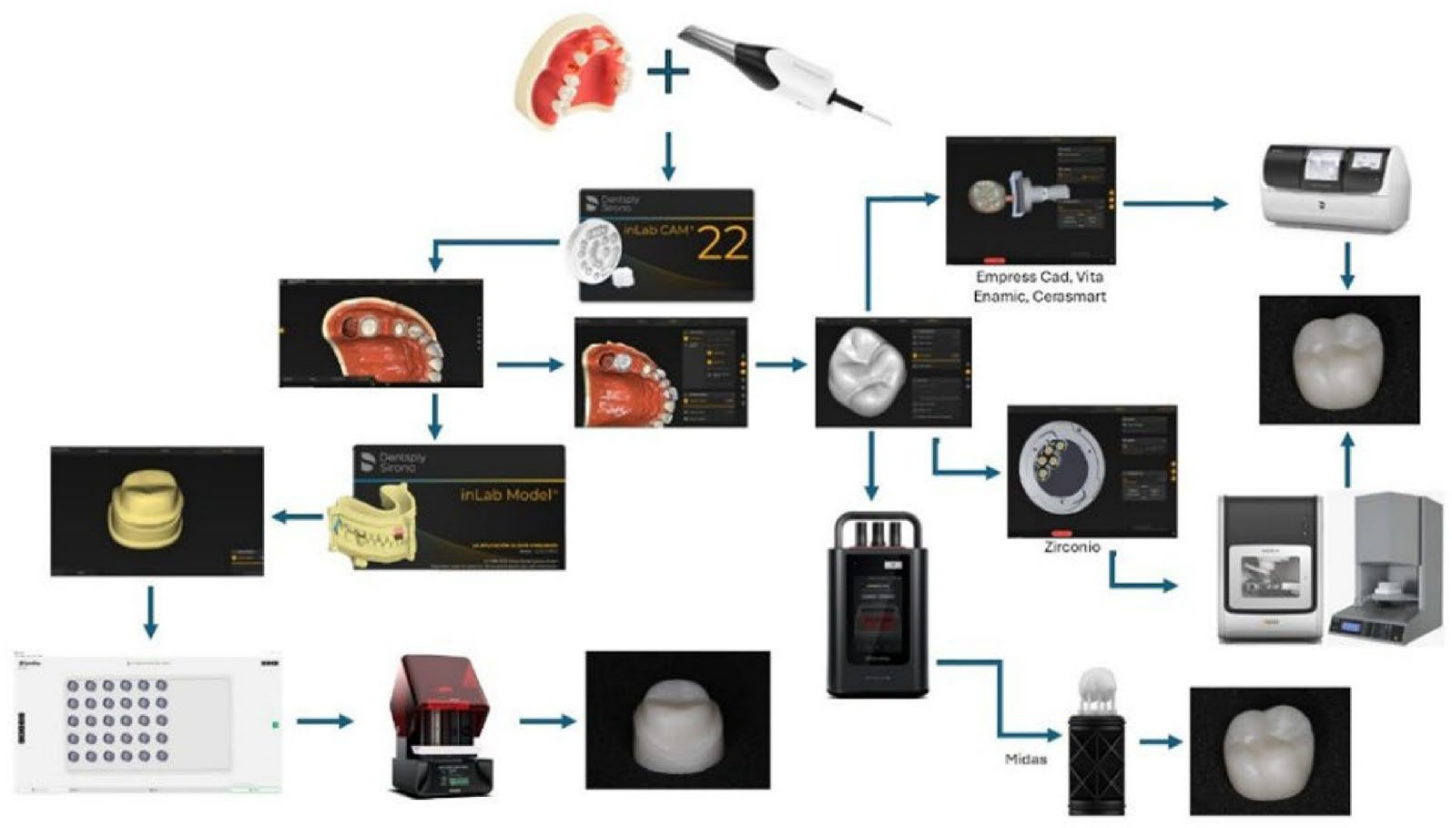
Schematic representation of the complete digital workflow used in the study. The additive group (MI) was fabricated using DPS technology (MIDAS; SprintRay, USA), including design, printing, post-processing, and polishing. The subtractive groups (CE, VE, EM) followed a conventional CAD/CAM protocol using a milling unit (PrimeMill; Dentsply Sirona, USA), with specific durations per material as indicated. All images are original and created by the authors

For the subtractive groups, the digital crown design was transferred to a milling unit (PrimeMill; Dentsply Sirona, USA) in “fine mode,” and post-processing was carried out according to the manufacturer’s guidelines. For the CE and VE materials, each crown was milled in 12 min and 34 s per block. In contrast, the EM material required 15 min per block (Fig. 1).

Ten zirconia crowns were milled using a five-axis milling machine (inLab MC X5; Dentsply Sirona, USA) over a total period of 104 min. The sintering process was carried out in a high-temperature furnace (InFire HTC; Sirona, Germany). The cycle began with an initial heating rate of 10 °C/min until reaching 900 °C, where the temperature was held for 30 min. Subsequently, the temperature was increased at a rate of 5 °C/min up to 1530 °C, where it was maintained for 120 min to complete the sintering process. Cooling was performed at a rate of 10 °C/min down to 900 °C, followed by a faster cooling phase at 15 °C/min until reaching 150 °C, concluding the cycle (Fig. 1).

Final polishing was performed using an extraoral polishing system (AP Polishing and Finishing Kit; Tampa, USA). The crowns were then seated on the 3D-printed resin models to verify their fit.

### Adhesive cementation protocol

**Fig. 2:**
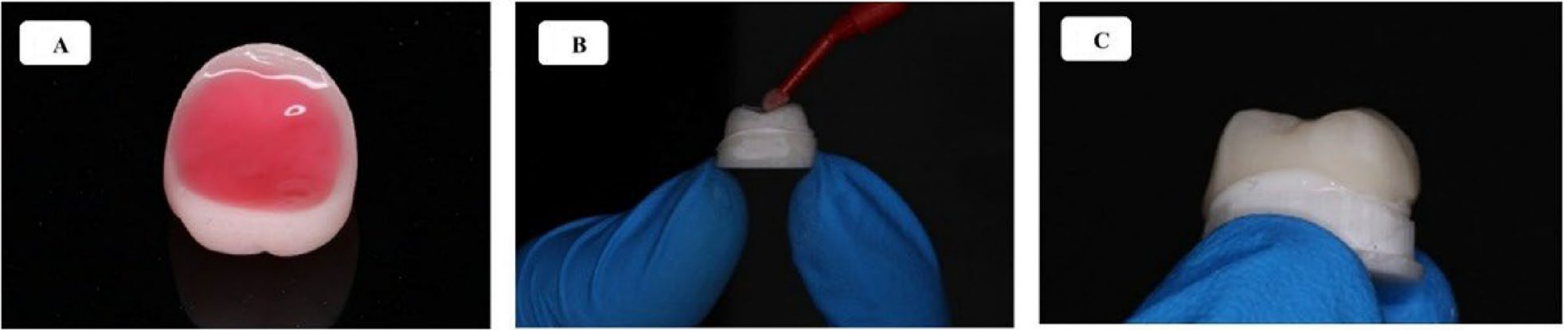
Representative images of the surface treatment and cementation protocol for the restorations. (2 A) Etching of hybrid material (Vita Enamic) with 9.8% hydrofluoric acid. (2B) Application of silane coupling agent to the abutment surface after sandblasting. (2 C) Seating of the crown over the prepared die with uniform pressure during cementation

 All restorations were adhesively luted following standardized procedures for both the abutment surfaces and the internal surfaces of the crowns. The surface treatment protocols were material-specific and are detailed in Table 3. Cementation procedures, including seating technique, light-curing, and final steps, were standardized across all groups and are summarized in Tables 3 and 4. Representative images of the conditioning and cementation steps are shown in Fig. 2 (Tables 3 and 4). All procedures—including digital scanning, CAD design, 3D printing, post-processing, sandblasting, and cementation—were performed by a single experienced operator trained in digital workflows and restorative techniques. The operator was previously calibrated through internal standardization protocols to ensure reproducibility and minimize inter-operator variability across all stages of the protocol.

**Table 3:** Summary of the surface conditioning protocols applied to each restorative material prior to adhesive cementation

Abutment Surface Treatment	Cementation Procedure
1. Sandblasting with 50 μm alumina oxide at 2 bar pressure for 10 s. and was standardized at a fixed distance of 10 cm.	1. Place the resin cement inside the crown.
2. Application of silane coupling agent for 20 min, then dry (Fig. 2B).	2. Seat the crown onto the die under constant pressure to ensure adaptation (Fig. 2C).
3. Application of dual-cure resin cement.	3. Remove excess cement using a No. 3 brush.
4. Application of AB adhesive (1:1 ratio), rub for 20 s, and air-dry.	4. Light-cure for 20 s per surface.
	5. Apply glycerin gel to the margins.
	6. Final light-curing for 40 s.

**Table 4:** Standardized cementation protocol used for all experimental groups. All procedures followed the manufacturer’s recommendations

Material	Conditioning Protocol
Vita Enamic	1. Etch with 9.8% hydrofluoric acid for 1 min; rinse and dry (Fig. 2A)
	2. Ultrasonic cleaning with alcohol for 5 min.
	3. Apply silane for 20 min and dry.
	4. Apply dual-cure resin cement.
Empress	1. Etch with 4.6% hydrofluoric acid for 60 s; rinse and dry.
	2. Ultrasonic cleaning with alcohol for 5 min.
	3. Apply silane for 20 s and dry.
	4. Apply dual-cure resin cement.
Cerasmart	1. Ultrasonic cleaning with alcohol for 5 min.
	2. Sandblast with 50 μm alumina at 2 bar pressure for 10 s.
	3. Apply silane for 1 min and dry.
	4. Apply dual-cure resin cement.
Crown MI	1. Ultrasonic cleaning with alcohol for 2–5 min.
	2. Sandblast with 50 μm alumina at 2 bar pressure for 10 s.
	3. Apply silane for 20 min and dry.
	4. Apply dual-cure resin cement.
Zirconia	1. Ultrasonic cleaning with alcohol for 5 min.
	2. Sandblast with 50 μm alumina at 2 bar pressure.
	3. Apply silane for 20 min and dry.
	4. Apply dual-cure resin cement.

Following cementation, all specimens were stored under controlled conditions for 24 h at room temperature to allow for complete polymerization. Subsequently, the samples were subjected to artificial aging through thermocycling to simulate oral environmental stresses. The thermocycling protocol is detailed below.

### Thermocycling and sample preparation for sectioning

The milled and printed crowns were separated by groups and placed into a custom basket with five compartments for aging through thermocycling. The samples were subjected to 5000 thermal cycles using a thermocycling device (Odome Dental Research – OMC 300 TS; Brazil), alternating between 5 °C and 55 °C in distilled water, with a dwell time of 25 s and a transfer pause of 10 s. Upon completion of the cycles, the samples were dried and fixed onto an acrylic base using thermoplastic resin to ensure stability.

### Sectioning and measurement of marginal and internal gaps

The crowns were sectioned using a precision cutting machine equipped with a diamond blade (IsoMet 1000; Buehler, Illinois, USA). Each crown was divided into four equal parts by performing two perpendicular cuts: the first in the buccopalatal direction, and the second in the mesiodistal direction, resulting in uniformly sized sections. To prevent deformation during sectioning—especially in the 3D-printed resin group—all specimens were embedded in acrylic resin blocks prior to cutting. Sectioning was performed using a precision diamond saw under constant water cooling and low-speed feed to minimize heat generation and mechanical stress. This protocol ensured clean, accurate cross-sections and preserved the dimensional integrity of each sample.

All final measurements were performed by a single operator with expertise in microscopic evaluation and digital measurement techniques. The operator was fully blinded to the group allocation of the specimens to prevent bias. This approach ensured consistency and objectivity in the data collection process.Gap measurements were performed using a digital optical microscope (AmScope T120B-M; AmScope, California, USA) at 40× magnification, connected to a digital camera. Measurements were acquired through the AmScope software (Version x64, 4.12.26598.20240928). Each section was stabilized on a microscope slide using sticky wax to ensure fixation during observation.

**Fig. 3:**
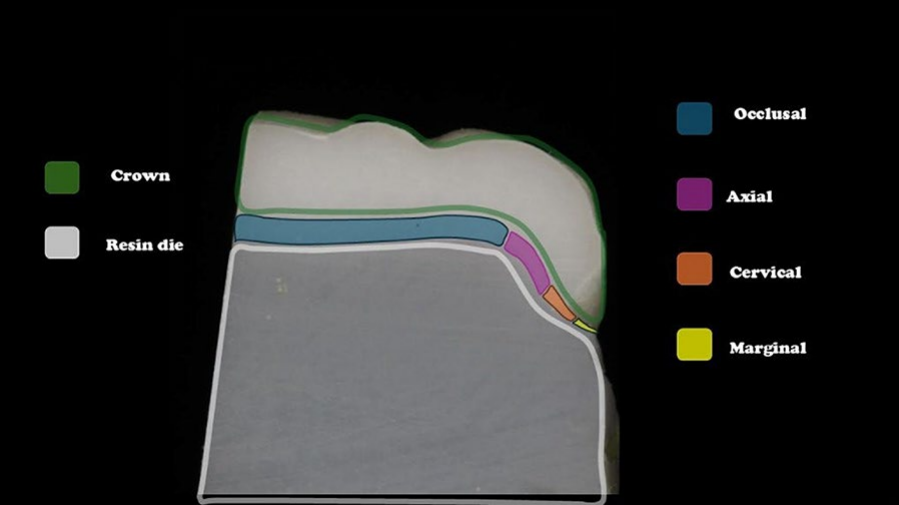
Schematic illustration of measurement zones for gap evaluation

For each section, both exposed surfaces were analyzed. On each surface, 20 measurements were taken: 5 at the marginal zone, 5 at the cervical third, 5 at the middle third, and 5 at the occlusal third, resulting in a total of 160 measurements per crown (Fig. 3).

Cross-sectional image of a sectioned crown cemented onto a resin die. The image identifies the measurement zones analyzed in the study: occlusal (blue), axial (purple), cervical (orange), and marginal (yellow).

### Statistical analysis

The normality of the gap dimension data (µm) was assessed using the Shapiro–Wilk test. Descriptive statistics, including mean, median, standard deviation, minimum, and maximum values, were calculated, and boxplots were generated for graphical representation. Due to the non-normal distribution of the data, comparisons between groups were conducted using the non-parametric Kruskal–Wallis test for independent samples. When significant differences were found, pairwise comparisons were performed using the Mann–Whitney U test. The level of statistical significance was set at 5% (*p* < 0.05). All analyses were performed using SPSS software (IBM SPSS Statistics, Version 27; IBM Corp., Armonk, NY, USA).

## Results

**Fig. 4:**
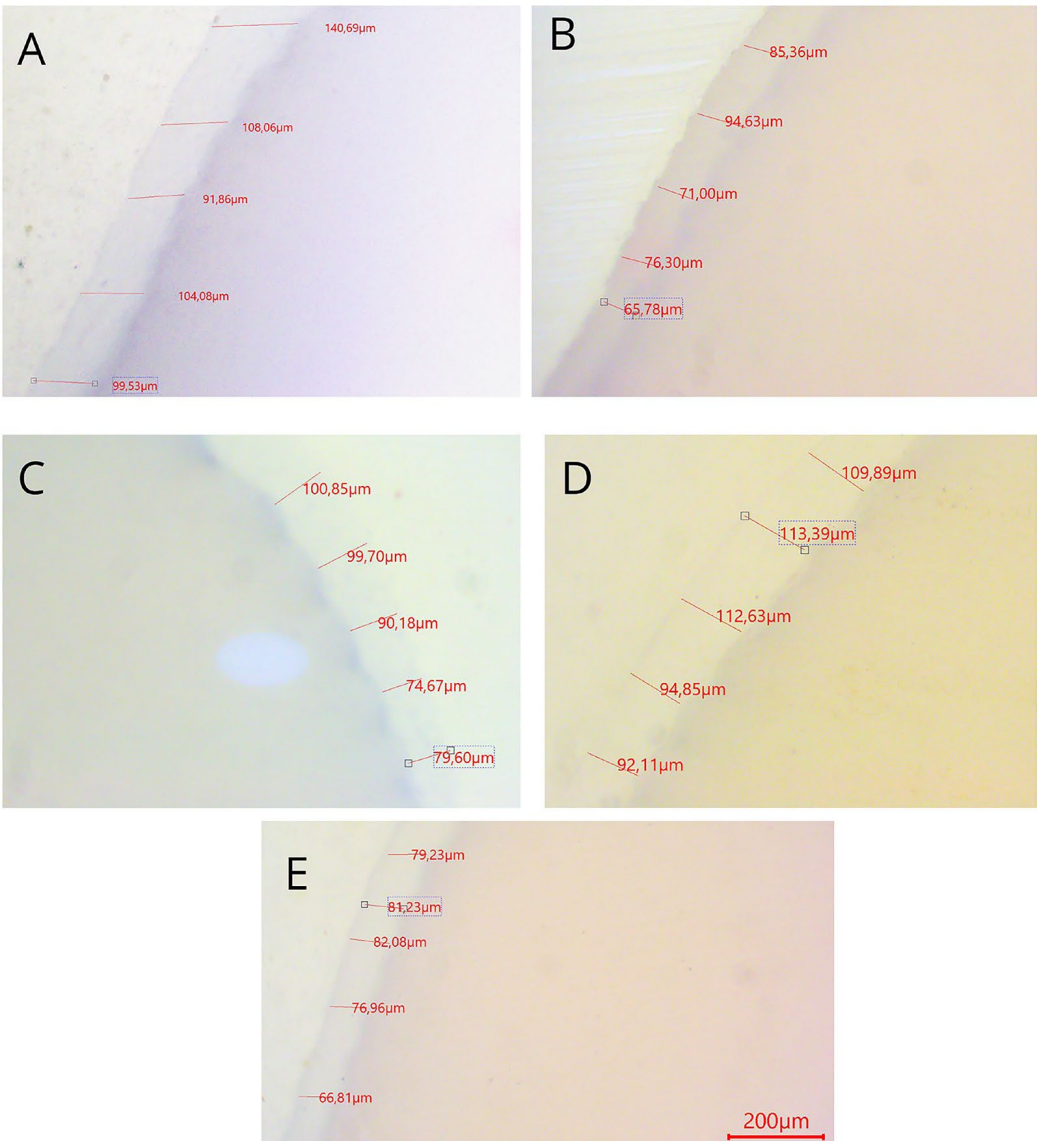
Gap measurement results under 40× magnification

Representative cross-sectional images showing gap dimensions (in µm) for the tested materials: (a) Empress CAD, (b) Zirconia, (c) Cerasmart, (d) Midas, and (e) Vita Enamic. Each image displays the adaptation between the crown and die in the occlusal, axial, cervical, and marginal zones.

Representative images of the marginal and internal gap dimensions obtained from the five restorative materials are shown in Fig. 4. Visual inspection under 40× magnification revealed qualitative differences in fit among materials, particularly at the marginal and occlusal zones.

**Fig. 5:**
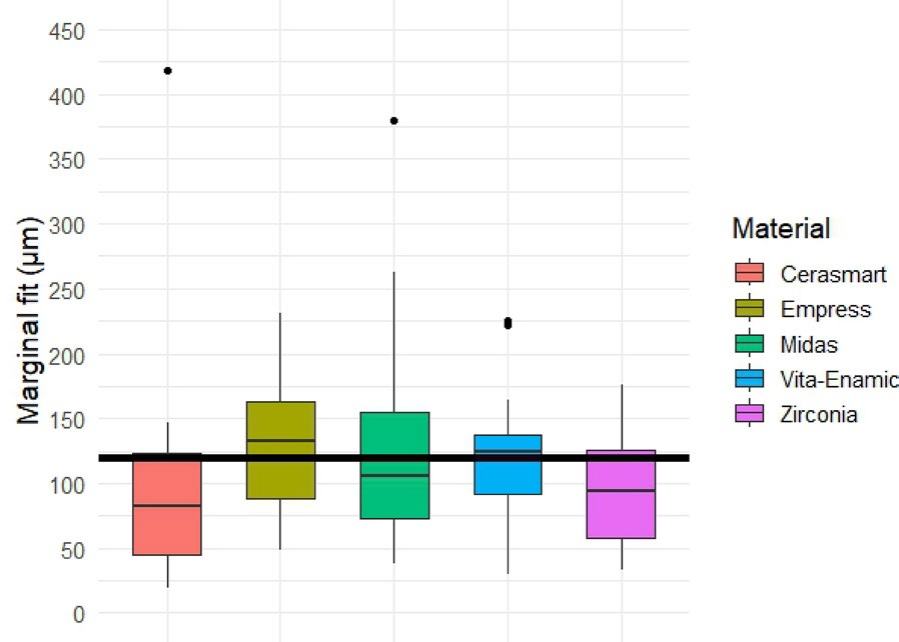
Box plot of marginal gap dimensions (in µm) for each restorative material. The boxes represent interquartile ranges (IQR), lines indicate medians, and whiskers show the full range of data. Statistically significant differences were observed between groups (*p* < 0.05). Black horizontal reference line represents 120 μm clinically acceptable threshold for gap dimensions

**Fig. 6:**
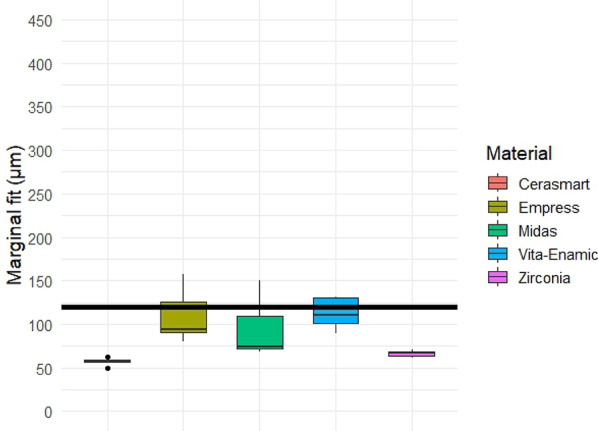
Box plot of cervical gap dimensions (in µm) for each restorative material. Significant differences were found among the groups (Kruskal–Wallis test, *p* = 0.001), with Cerasmart and zirconia showing the smallest gaps and lowest variability. Black horizontal reference line represents 120 μm clinically acceptable threshold for gap dimensions

**Fig. 7:**
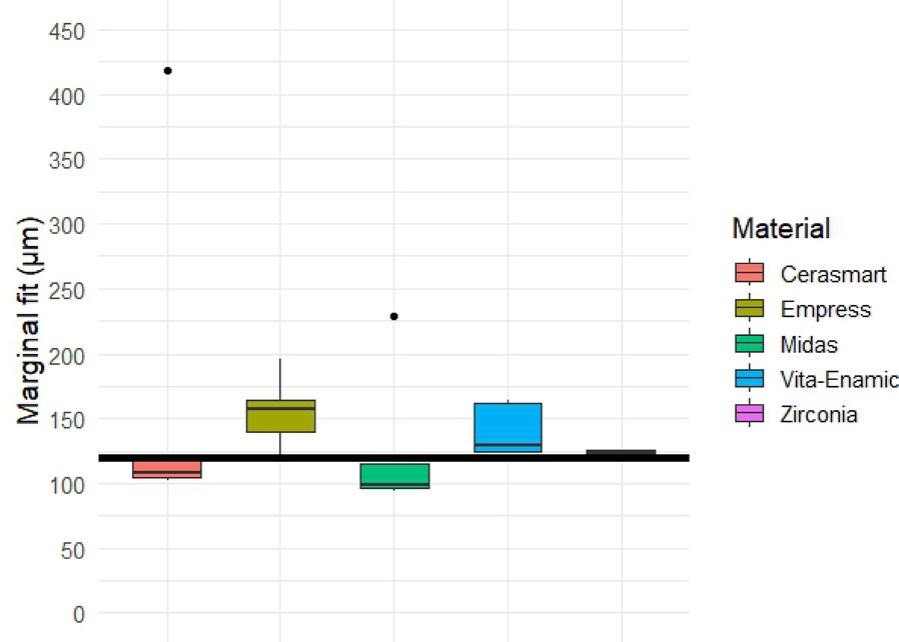
Box plot of axial gap dimensions (in µm) for each restorative material. No statistically significant differences were found among the groups (Kruskal–Wallis test, *p* = 0.0717). Zirconia showed the lowest variability and most homogeneous measurements. Black horizontal reference line represents 120 μm clinically acceptable threshold for gap dimensions

**Fig. 8:**
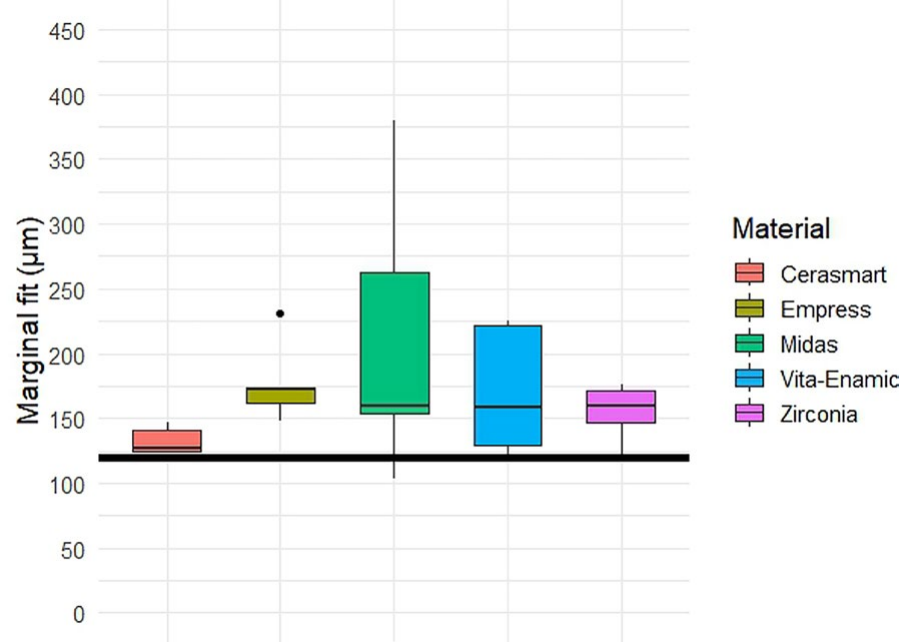
Box plot of occlusal gap dimensions (in µm) for each restorative material. Although Cerasmart exhibited the lowest gaps and variability, no statistically significant differences were observed among the groups (Kruskal–Wallis’s test, *p* = 0.1749). Black horizontal reference line represents 120 μm clinically acceptable threshold for gap dimensions

Quantitative analysis of the gap dimensions was performed in four anatomical zones: marginal, cervical, axial (middle third), and occlusal. Box plot distributions for each zone are presented in Figs. 5, 6, 7 and 8, and descriptive statistics are summarized in Table 5. Overall, significant differences were observed between materials in all regions (*p* < 0.05).

Marginal gap. Box plot analysis of the marginal gap dimensions is shown in Fig. 5. The lowest median marginal gap was observed in the zirconia group, followed closely by Empress CAD. The Midas and Cerasmart groups showed higher median values, while Vita Enamic presented the widest interquartile range and highest variability. Statistically significant differences were found among the groups (Kruskal–Wallis test, *p* < 0.05), with post-hoc pairwise comparisons (Mann–Whitney U test) revealing significant differences particularly between zirconia and hybrid materials.

**Table 5:** Descriptive statistics of marginal gap measurements (in µm) for each restorative material

Statistic	Empress CAD	Vita Enamic	Cerasmart	Midas	Zirconia
Mean (µm)	127.4	121.7	96.6	132.8	95.9
Median (µm)	133.1	124.1	82.2	106.3	94.1
Standard Deviation (µm)	51.4	50.7	87.1	86.3	47.9
Coefficient of Variation (%)	40.4%	41.7%	90.2%	65.0%	49.9%
Minimum (µm)	48.3	29.2	19.0	37.6	32.7
Maximum (µm)	231.3	225.7	418.0	379.7	175.6
p-value (Kruskal–Wallis)	0.0716				

Box plot analysis of cervical gap measurements is presented in Fig. 6, and descriptive statistics are summarized in Table 6. Cerasmart and zirconia showed the lowest mean and median values, with zirconia also exhibiting the lowest variability (coefficient of variation: 6.1%). Empress CAD and Midas presented wider distributions and higher gap dimensions. The Kruskal–Wallis test revealed statistically significant differences among groups (*p* = 0.001). Post-hoc pairwise comparisons showed significant differences particularly between zirconia or Cerasmart and the other materials.

**Table 6:** Descriptive statistics of cervical gap measurements (in µm) for each restorative material

Statistic	Empress CAD	Vita Enamic	Cerasmart	Midas	Zirconia
Mean (µm)	109.5	112.4	56.9	94.6	66.0
Median (µm)	93.7	110.9	57.8	73.8	67.1
Standard Deviation (µm)	31.9	18.5	4.7	35.1	4.0
Coefficient of Variation (%)	29.1%	16.5%	8.3%	37.1%	6.1%
Minimum (µm)	79.6	89.0	49.5	68.1	60.8
Maximum (µm)	157.5	131.2	62.2	149.8	70.6
p-value (Kruskal–Wallis)	0.001				

### Axial gap

The comparison of axial gap dimensions between restorative materials did not reveal statistically significant differences (*p* ≥ 0.05). Among all groups, the zirconia specimens exhibited the lowest mean axial gap (122.7 μm), along with the smallest variability (coefficient of variation: 2.9%), indicating greater consistency and homogeneity among measurements (Table 7; Fig. 7).

**Table 7:** Descriptive statistics of axial gap dimensions (in µm) for each restorative material

Statistic	Empress CAD	Vita Enamic	Cerasmart	Midas	Zirconia
Mean (µm)	155.0	141.0	171.1	126.8	122.7
Median (µm)	157.3	129.6	108.6	98.9	123.4
Standard Deviation (µm)	29.1	20.7	138.2	57.4	3.5
Coefficient of Variation (%)	18.8%	14.7%	80.8%	45.3%	2.9%
Minimum (µm)	117.4	124.1	102.3	94.6	117.7
Maximum (µm)	196.0	164.9	418.0	228.3	126.1
p-value (Kruskal–Wallis)	0.0717				

Oclusal gap measurements are summarized in Table 8 and illustrated in Fig. 8. The Cerasmart group exhibited the lowest mean and median values, with minimal variability (coefficient of variation: 8.0%). In contrast, Midas showed the highest mean gap (211.9 μm) and the widest distribution (CV: 52.1%). However, the differences among materials were not statistically significant (*p* = 0.1749, Kruskal–Walli’s test).

**Table 8:** Descriptive statistics of occlusal gap dimensions (in µm) for each restorative material

Statistic	Empress CAD	Vita Enamic	Cerasmart	Midas	Zirconia
Mean (µm)	177.6	171.5	132.5	211.9	155.2
Median (µm)	172.4	158.3	127.3	160.1	159.7
Standard Deviation (µm)	31.8	49.7	10.6	110.4	21.5
Coefficient of Variation (%)	17.9%	29.0%	8.0%	52.1%	13.9%
Minimum (µm)	147.6	122.0	122.8	102.9	122.4
Maximum (µm)	231.3	225.7	146.3	379.7	175.6
p-value (Kruskal–Wallis)	0.1749				

### Overall gap comparison

**Fig. 9:**
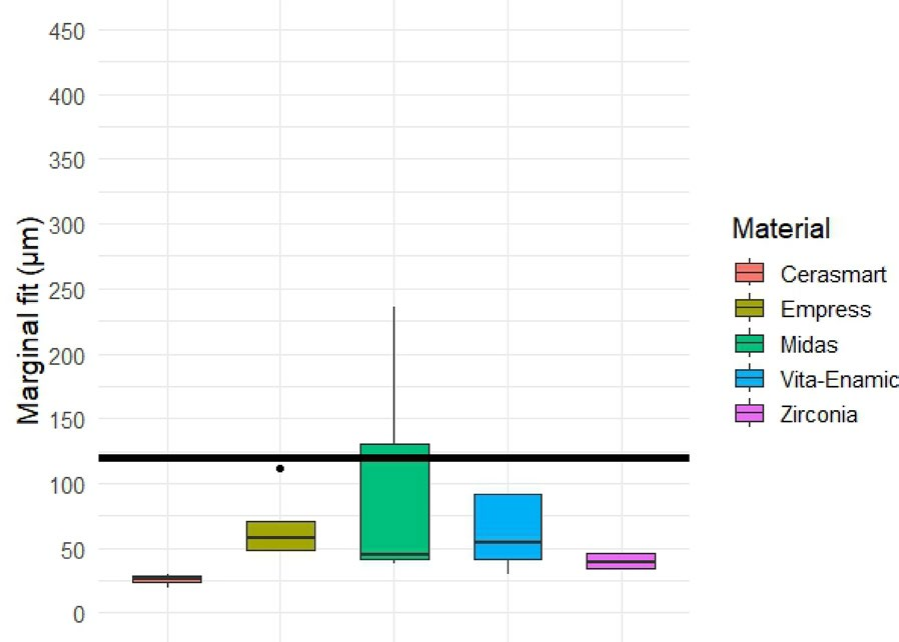
Box plot showing the overall gap dimension distribution (in µm) for each restorative material, based on all measurement zones combined (marginal, cervical, axial, and occlusal). Cerasmart exhibited the smallest gaps with minimal variability, significantly different from Empress CAD and Midas (*p* = 0.0063). Black horizontal reference line represents 120 μm clinically acceptable threshold for gap dimensions

The global analysis of gap dimensions across all measured zones is summarized in Table 9; Fig. 9. Statistically significant differences were observed between restorative materials (*p* = 0.0063, Kruskal–Wallis test), with post-hoc comparisons revealing that Cerasmart exhibited the lowest overall gap values, significantly different from Empress CAD and Midas. Zirconia showed intermediate performance with low variability, while Midas displayed the widest distribution. These findings reflect the combined behavior of each material across the marginal, cervical, axial, and occlusal regions.

**Table 9:** Overall gap dimension summary (µm) for each restorative material across all measurement zones. Different superscript letters (A, B) indicate statistically significant differences between groups (*p* = 0.0063, Kruskal–Wallis with post-hoc Mann–Whitney U

Statistic	Empress CAD	Vita Enamic	Cerasmart	Midas	Zirconia
Mean (µm)	67.5 A	61.8 A	25.7 B	97.8 A	39.7 AB
Median (µm)	57.9	54.3	26.6	44.6	39.0
Standard Deviation (µm)	26.6	29.0	4.4	86.3	6.3
Coefficient of Variation (%)	39.4%	46.9%	17.1%	88.2%	15.9%
Minimum (µm)	48.3	29.2	19.0	37.6	32.7
Maximum (µm)	112.2	92.3	30.0	235.8	46.4
p-value	0.0063				

Different superscript letters (A, B) indicate statistically significant differences between groups (*p* = 0.0063, Kruskal–Wallis with post-hoc Mann–Whitney U

## Discussion

This study compared the marginal, cervical, axial, and occlusal gaps of full-coverage crowns fabricated using various CAD/CAM restorative materials. Four materials were processed using a conventional subtractive (milling) workflow (control group), while one experimental group (MI) was fabricated using a recently introduced additive manufacturing system based on Digital Press Stereolithography (DPS). No statistically significant differences were found in most regions; therefore, the null hypothesis was accepted.

Although the differences were not statistically significant, the quantitative analysis showed that zirconia consistently exhibited the lowest gap values across nearly all measurement zones (cervical, axial, occlusal, and marginal). It also had the lowest coefficient of variation, indicating high precision and measurement consistency. Specifically, in the axial region, zirconia presented the smallest gap (122.7 μm) with minimal variability (CV = 2.9%), reflecting strong homogeneity among samples.

The new MI material exhibited the highest marginal gap mean (132.8 μm) and a high degree of variability (CV = 65.0%), suggesting limited consistency in cervical and axial regions. While its mean values in those zones were intermediate (94.6 μm and 126.8 μm, respectively), the elevated variability indicates reduced reliability. In the occlusal region, MI showed the highest mean gap (211.9 μm) and the widest dispersion (CV = 52.1%). In the marginal zone, it also exhibited the highest overall mean (97.8 μm) and the highest variability (CV = 88.2%).

Although not always statistically significant, the performance of MI was comparable to that of Empress CAD, slightly better than Vita Enamic, and inferior to Cerasmart and zirconia. The increased gap observed in the occlusal zone may be associated with cementation using a higher-viscosity resin cement. In the context of a 3D printing system, these findings suggest the need for further investigation, particularly focusing on occlusal gap adaptation.

The observed differences in marginal gap values between milled and 3D-printed restorations may be influenced by multiple factors inherent to each manufacturing technique and their respective post-processing protocols [26]. Milled restorations fabricated using a high-performance compact milling unit (PrimeMill; Dentsply Sirona, Germany) tend to exhibit superior dimensional accuracy due to the mechanized precision of the subtractive process and the structural stability of pre-sintered industrial blocks.

In the case of zirconia, the material is milled in its pre-sintered (soft) state using a five-axis milling system, which may partly explain its consistency and low variability in gap measurements. However, it is important to note that zirconia undergoes volumetric shrinkage during the sintering stage, which can potentially affect marginal accuracy if not properly compensated for during the digital design process [27]. Errors during the cementation procedure—such as non-uniform cement distribution, inadequate seating pressure, or inconsistent working times—can lead to discrepancies in the final fit. However, these issues were not visually evident in this study [28]. Three-dimensional (3D) printed restorations are subject to specific limitations related to additive manufacturing, including printing resolution, build orientation, layer thickness, and potential deformation during photopolymerization and post-curing, all of which may compromise marginal adaptation [29]. These factors may have contributed to the minor differences observed between printed and milled restorations.

Lerner (2021) reported superior marginal fit in monolithic zirconia crowns compared to 3D-printed alternatives [30]. Similarly, Ates (2016) highlighted that factors such as ceramic crystallization and sintering, cementation technique, milling parameters, and measurement methodology all influence marginal precision [31]. Yilbas (2024) supported this view, noting that analog printing and light-curing protocols in zirconia yield better marginal fit [32]. Additionally, Akbar (2006) found that shoulder finish lines resulted in more favorable marginal adaptation (15–78.7 μm) compared to chamfer margins (12.2–256 μm), suggesting that horizontal cervical designs provide more predictable and uniform fits [33].

In our study, all materials demonstrated marginal adaptation averages ranging from 88 μm in the zirconia group to 142 μm in the Empress group. MI presented an intermediate value of 113 μm, which is within the clinically acceptable range when combined with a horizontal cervical finish line.

Reich (2025) reported that acceptable marginal gap values for ceramic restorations range between 64 and 84 μm [34], while Baig et al. (2022) suggested a clinical threshold of 120 μm for crowns fabricated with zirconia and lithium disilicate [35]. These findings align with Del Piña’s (2021) study on ceramic crowns, which reported marginal gaps ranging from 3 to 174 μm [36]. Laumbacher (2022) found acceptable discrepancies between 15 and 120 μm in milled zirconia restorations, and documented marginal gaps as low as 7.6 μm and as high as 206.3 μm in monolithic crowns [37].

In the present study, all ceramic materials demonstrated marginal gap values within the clinically acceptable range, ranging from 95.9 μm (zirconia) to 132.8 μm (MI). Suksuphan et al. (2023) evaluated the marginal fit of four materials—Vita Enamic, Grandio Bloc, lithium disilicate, and zirconia—and reported the highest precision in zirconia (55.95 μm), followed by Vita Enamic (116.36 μm) [38]. These results are consistent with our findings, where zirconia showed superior marginal adaptation (95.9 μm), and Vita Enamic presented values of 121.7 μm. This supports the notion that zirconia remains one of the most reliable materials for indirect restorations in terms of marginal fit.

Al-Humood et al. (2023), in a systematic review of 19 studies comparing printed and milled restorations, concluded that although 3D-printed restorations showed slightly higher discrepancies, the differences were not statistically significant [39]. Similarly, Refaie (2023) and Alghauli (2024) found that printed crowns exhibited higher marginal gaps (80 ± 30 μm) compared to milled zirconia crowns (60 ± 20 μm), but both remained within the clinically acceptable range of 50–120 μm [40, 41]. These studies corroborate our findings, indicating that while milled crowns tend to have superior adaptation, 3D-printed restorations are clinically viable.

Recent evidence has suggested that the clinical performance of 3D-printed restorations may approach that of milled materials under certain conditions. Valenti (2024) reported that polymeric printed materials exhibit mechanical and marginal properties comparable to their milled counterparts, recommending their use in provisional and short-span prostheses [42]. This aligns partially with our findings, in which the MI-printed crowns achieved marginal gaps within the clinically acceptable range.

However, our data also revealed that MI demonstrated higher variability, particularly in the cervical and occlusal regions, suggesting that printed materials may still be more sensitive to manufacturing inconsistencies. Although some studies, such as those by Kakinumay (2022) and Khwanpuang (2024), reported superior marginal fit in printed restorations [43, 44], our results do not fully support this, especially when compared to the more consistent performance observed in zirconia and Cerasmart.

Zhu (2024) cautioned against the widespread clinical use of current 3D printing materials, citing insufficient maturity for long-term applications [45]. Our findings agree in part, as we observed acceptable average values for MI, but also noted high variability, raising concerns about reliability in more demanding clinical scenarios.

On the other hand, Duarte (2025) highlighted that integrating ceramic fillers homogeneously into the polymer matrix is key to improving the behavior of printed restorations [46]. The MI system, with its capsule-based DPS approach, may represent a step forward in this direction. Our results suggest that MI can produce clinically acceptable restorations rapidly, which is particularly relevant in chairside workflows. Nonetheless, its higher inconsistency compared to milled materials like zirconia suggests that further optimization is still required.

In previous studies, Vita Enamic (VE) has shown favorable marginal adaptation. Oguz (2020) reported lower marginal discrepancies in VE (39.79 μm) compared to Cerasmart (47.41 μm) using full-surface digital alignment techniques [47]. Naffah (2019) similarly found that VE exhibited superior marginal fit compared to CE, with both remaining within the clinically acceptable range of 120 μm. Furthermore, the same study noted that Empress CAD (EM) demonstrated acceptable adaptation, although it did not outperform CE in terms of precision or dimensional stability [48].

In contrast, our findings suggest a different performance hierarchy. Cerasmart exhibited the lowest average marginal gap among all tested materials (96.6 μm), followed closely by zirconia. VE displayed slightly higher values (121.7 μm), while EM showed the largest average gap (127.4 μm). These results do not fully align with prior findings favoring VE over CE, and may be influenced by differences in study design, sample size, measurement protocols, or cementation procedures. Notably, the greater variability observed in EM could reflect material-specific sensitivity to milling or cementation parameters, rather than intrinsic inferiority.

Gold et al. reported lower and more consistent marginal gap values for EM (49.2 ± 5.5 μm) [49], which differ substantially from the averages observed in our study. Such discrepancies highlight the influence of methodological variables—including the type of scanner, software calibration, and evaluator technique—on gap measurement outcomes. Despite the variation in reported values across the literature, the marginal gaps recorded in our study for all three materials remained within clinically accepted thresholds, suggesting that each remains a viable restorative option.

Although no statistically significant differences were found in the marginal, axial, and occlusal zones (*p* > 0.05), the descriptive statistics revealed clinically meaningful trends. Zirconia consistently demonstrated the lowest mean gap values and the smallest coefficient of variation across all regions, indicating superior dimensional stability. Conversely, Midas exhibited higher variability, particularly in the occlusal and cervical zones, which may reflect sensitivity to post-processing or cementation parameters. These findings underscore the importance of evaluating not only statistical significance but also the clinical relevance and consistency of restorative materials. To our knowledge, this is one of the first in vitro investigations to quantitatively assess marginal, cervical, axial, and occlusal gap dimensions in full crowns fabricated with the MI-DPS system. These findings provide initial benchmark data and may guide future research on the clinical viability of this emerging technology.

From a clinical perspective, marginal and internal adaptation are essential parameters that influence the long-term success of full-coverage restorations. Poor adaptation has been associated with microleakage, secondary caries, loss of retention, and decreased mechanical performance. The results of this study demonstrate that, although the MI-DPS system exhibited higher variability compared to milled materials, it maintained gap values within clinically acceptable thresholds. This suggests that such 3D-printed resins may represent a viable alternative for definitive restorations—especially in workflows where speed, material efficiency, and digital integration are critical. However, clinical decisions should still be guided by case selection, proper post-processing, and further validation under intraoral conditions.

This study has several limitations that must be acknowledged. As an in vitro investigation, the results cannot be fully extrapolated to intraoral conditions. Factors such as cement application, mixing protocol, and the pressure applied during cementation—although standardized and performed by a calibrated prosthodontist—could still introduce variability. Furthermore, the marginal gap measurements were conducted manually using image analysis software and a micrometric calibration scale, which may introduce observer-dependent bias.

Although this study focused exclusively on the marginal and internal adaptation of restorations, we acknowledge that other material properties—such as mechanical strength, aging resistance, and dimensional stability—are equally important for clinical success. Future research should address these aspects to provide a more comprehensive assessment of the clinical viability of ceramic-filled 3D-printed resins like MI-DPS.

A potential limitation of the present study is the restricted number of 3D-printed materials evaluated. While multiple subtractive materials of varying compositions were included for comparison, only one 3D-printed resin—fabricated with the MI-DPS system—was analyzed. This choice was based on the novel nature of the DPS technology and its specific indication for definitive restorations using high-viscosity, high-filler content resins. At the time of experimentation, few commercially available 3D-printing systems offered equivalent clinical indications. However, we acknowledge that the inclusion of additional 3D-printed resins could have broadened the comparative scope and offered a more comprehensive evaluation of additive manufacturing technologies. Future research should investigate and compare multiple 3D-printed materials under standardized conditions to validate and expand upon these preliminary findings.

Another important consideration is the role of post-processing in 3D-printed restorations. The MI system, despite its promising speed and material properties, may be particularly sensitive to variations in washing, curing, or handling steps. Manual sectioning of samples using a precision saw, although standardized, may also affect measurement accuracy due to possible deviations in angulation or alignment. It is also important to acknowledge that the tested 3D-printed resin presents fundamental differences in composition and polymerization behavior when compared to conventional CAD/CAM blocks, which are industrially processed under high temperature and pressure. These intrinsic differences—particularly in filler content, matrix structure, and post-curing dynamics—may influence internal adaptation and dimensional stability. While our study provides a first independent evaluation of this material, direct comparisons with well-established milled materials must be interpreted with caution. Further clinical and laboratory investigations are necessary to fully understand the implications of these compositional differences.

Although resin-based dies were used to simulate the elastic modulus of natural teeth, clinical trials remain necessary to validate these findings under real-world conditions. The sample size of ten specimens per group was based on protocols commonly reported in the literature for marginal fit studies, and was considered adequate due to the high number of repeated measurements (160 per crown), which increases statistical reliability. Additionally, future studies should investigate the influence of cement viscosity, cementation pressure, and restoration geometry—particularly in relation to occlusal gap formation—in printed restorations fabricated with novel technologies such as Digital Press Stereolithography (DPS).

## Conclusions

Within the limitations of this in vitro study, the following conclusions can be drawn:

- All tested CAD/CAM materials—including both subtractively milled and additively printed systems—demonstrated marginal, cervical, axial, and occlusal gap values within clinically acceptable thresholds for full-coverage crowns.
- Zirconia consistently showed the lowest gap values and the highest dimensional stability, particularly in the axial and cervical regions, confirming its reliability as a restorative material for precise adaptation.
- The MI-DPS 3D printing system exhibited greater variability across all measurement zones compared to milled materials, especially in occlusal and cervical areas. Nonetheless, its average marginal gap (132.8 μm) remained within accepted clinical limits.
- Cerasmart and Vita Enamic demonstrated favorable adaptation with moderate variability, whereas Empress CAD showed the highest average gaps, though still within the acceptable range.
- Although this study provides an initial independent evaluation of the MI-DPS 3D printing system for crown fabrication, its clinical applicability should be interpreted with caution. The current results are limited to marginal and internal fit; further studies assessing physical and mechanical properties are necessary before definitive clinical conclusions can be drawn.
- Additional in vitro and in vivo research is encouraged to validate these findings under intraoral conditions and to further explore the performance of emerging additive manufacturing materials such as MI-DPS.

.

## Data Availability

No datasets were generated or analysed during the current study.
